# Asylum Schools for the Insane

**Published:** 1870-12

**Authors:** 


Asylum Schools for the Insane.—Several of the public institutions of Europe have for many years past had, to their ordinary treatment of lunatics, the addition of a well-ordered school, in which patients are instructed in the various fundamental branches of a common school education, as in any of our public schools. This practice has never been introduced into the American asylums.

The system, by those who practice it, is said to work admirably. Of course it is not expected that lunatics will deport themselves or make progress in their studies like sane persons, but they are found to invariably take to the school exercises, not merely with willingness, but with real enthusiasm; and it furnishes a diversion for the mind, a new exercise with which to amuse and entertain patients, which is found very beneficial to the particular class of invalids.

As might be expected, the amount of real knowledge gained by these people, to be carried with them when discharged, is small; but the object of the system is more to amuse, divert, and exercise the mind of the patient; it is an exercise, by the use of which only, says one of the superintendents, "what may be called the *moral treatment* can be fully carried out."—*American Journal of Insanity.*

